# Delayed diagnosis of a case of Zenker’s diverticulum. What may happen when a family physician does not visit his family physician

**DOI:** 10.1080/13814788.2018.1464556

**Published:** 2018-05-04

**Authors:** Zekeriya Akturk, Ali Bilal Ulas, Atila Eroglu

**Affiliations:** a Family Physician, ailem Academic Counseling, Izmir, Turkey;; b Department of Thoracic Surgery, Ataturk University Medical Faculty, Erzurum, Turkey

**Keywords:** Technology and medicine, Zenker’s diverticulum, gastroesophageal reflux disease

## Abstract

**Introduction:** Zenker’s diverticulum is a diverticulum of the mucosa of the pharynx, just above the cricopharyngeal muscle. It occurs commonly in elderly patients (over 70 years) and the typical symptoms include dysphagia, regurgitation, chronic cough, aspiration and weight loss.

**Case:** We are reporting dysphagia in a 49-years old man who was treated as having *Helicobacter Pylori* gastritis for three years. Being a family physician himself, the patient applied to specialists in gastroenterology, bypassing primary care. During a casual interview on his symptoms, a family physician referred him to undergo a repeated endoscopy with suspected Zenker’s diverticulum. After being diagnosed with Zenker’s diverticulum, the patient underwent surgical intervention at the department of thoracic surgery and made a full recovery. He regained five kilograms at the end of five weeks after the operation.

**Conclusion:** This case demonstrates once more the importance of history taking and follow-up in medical care. Attentive listening by a family physician could have probably prevented the delay of service in this case.

KEY MESSAGES

- The incidence of Zenker’s diverticulum is around 1–2 per 100,000 patients/year.
- Some symptoms of Zenker’s diverticulum may resemble gastroesophageal reflux disease (GERD).
- Today’s general practitioners must apply a high-tech/high-touch oriented medical model.

## Introduction

The pharyngeal pouch or Zenker’s diverticulum (ZD) is a diverticulum of the mucosa of the pharynx. It occurs commonly in elderly patients, and the typical symptoms include dysphagia, regurgitation, chronic cough, aspiration, and weight loss [1].

The incidence of ZD is estimated to be 1–2 per 100,000 patients/year and twice as prevalent in males [2]. Its exact aetiology remains unclear. However, the most accepted theory is that the disease is consequent to a functional disorder of pharyngo-oesophageal motility, represented by an increased resting pressure of the upper oesophageal sphincter, lack of relaxation during swallowing, and especially the lack of synchronization between upper oesophageal sphincter and hypopharynx during swallowing [3]. Relaxation and early closing of the upper oesophageal sphincter during swallowing may contribute to the development of ZD [4].

ZD manifests itself with characteristic symptoms and signs. The first and most commonly reported symptom is gradually increasing dysphagia (80–90% of cases) followed by regurgitation (60%) and cough (30–40%). As a consequence of regurgitations, the aspiration of ingesta into the bronchial tree may appear. Characteristics are loud swallowing of liquids, Boyce’s sign (a noise of splashing fluid accumulated in the diverticulum), cough, and hoarseness. Over time, the diverticulum grows, and patients complain of dysphagia due to oesophagus constriction by the filled diverticulum. Voice alteration and halitosis may also occur [5].

Due to the typical presenting history and symptoms, ZD should be easily suspected by family physicians [6]. However, the final diagnosis is based on endoscopy and a radiogram with barite [7].

We present a case of ZD, who was mistreated as gastroesophageal reflux for three years.

## Case

The patient is a 49-year-old, 163 cm-tall man, a professor of family medicine, who was teaching at a university hospital when a dysphagia and night cough started to bother him. Although the patient himself would be expected to advise others to first see a family physician for this kind of symptoms, he decided to use specialized care services at the university hospital he was working for in April 2015. Acquainted with the patient, the professor of gastroenterology ordered an immediate oesophagogastroduodenoscopy, which was reported as ‘hiatal insufficiency and antral gastritis’. The gastric biopsy specimen taken during the endoscopy session returned positive for *Helicobacter pylori.*

The patient was diagnosed as having gastroesophageal reflux disease (GERD) and prescribed a scheme of amoxicillin + clarithromycin + pantoprazole + bismuth subsalicylate. After a few days, he returned to his doctor complaining of medication side effects such as burning in his throat and oesophagus and not being able to swallow the pills. However, being warned of the consequences of non-compliance with the treatment, he kept using the medications as advised. There was no improvement in the initial symptoms at the end of the 14-days treatment. The patient was instructed to continue using pantoprazole 40 mg/day for another three months. However, he discontinued the medication after few weeks due to no apparent benefit.

The patient did not seek any other formal health advice for the next three years. He kept taking pantoprazole for a few days when his symptoms flared up. With time, he was used to his symptoms and learned to manage his condition by lifestyle changes such as eating less, chewing longer than usual, and arranging his sleep position. During an informal discussion with a family physician in June 2017, he was advised to see his family doctor, who took a thorough history, which revealed the following:

- The patient was reporting rare (once every three-to-four weeks) night coughs for more than 20 years. Initially, the cough was awakening from sleep but usually not reoccurring for a long time.
- His primary symptoms of the current episode of his disease started at the end of 2014, after a stressful event leading to a loss of his job.
- The principal symptom was dysphagia and a sense of fullness in his throat, which he described as ‘Having a bread loaf in his oesophagus’.
- He did not report any retrosternal burning or acid reflux.
- He usually woke every night with persistent cough attacks lasting 30–40 min. The cough increased in cold weather and sleeping in the supine position.
- Other exacerbating factors were drinking tea or coffee, consuming too hot or too cold food, as well as swallowing large mouthfuls of food.
- He was reporting undigested food coming back up into his mouth from the oesophagus, even one-to-two hours after a meal.
- There was a typical gurgling sound while swallowing, especially when drinking liquids.
- Recently (two-to-three months) he started to experience saliva oozing from his mouth onto the pillow while asleep. He needed to have a napkin ready to wipe his mouth when awoke.
- The patient reported a weight loss of 16 kg within the last year, dropping from 74 kg to 58 kg.

**Figure 1.**
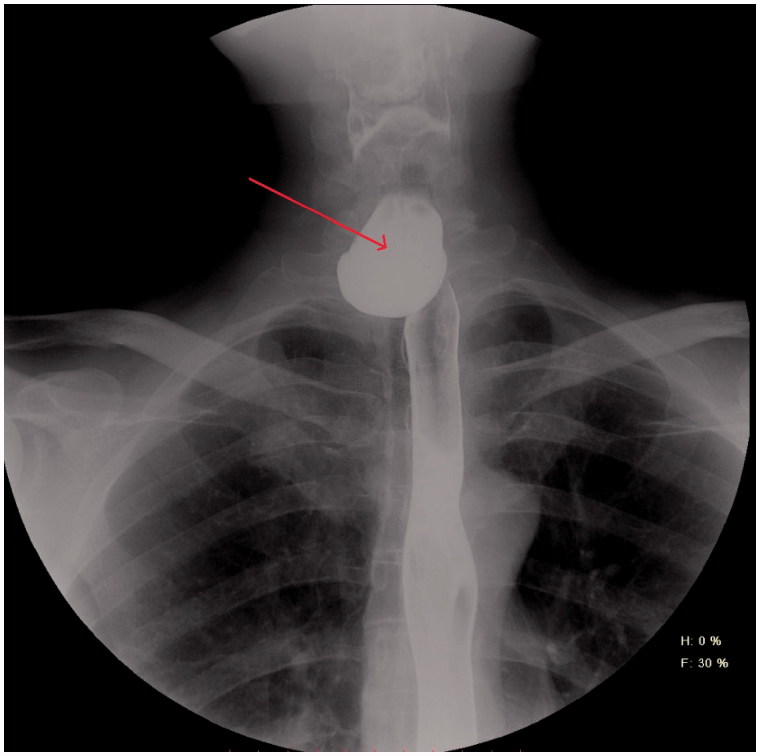
Contrast oesophagoscopy, postero-anterior view.

His family physician referred the patient to another gastroenterologist with the differential diagnosis of ZD. This time, the endoscopy report was positive for ZD, describing a significant diverticular opening at the left posterior side of the oesophagus, at around 18 cm from the teeth, which initiated a further referral to the department of thoracic surgery. Contrast oesophagoscopy (Figure 1) revealed a large ZD.

The patient was admitted to the department of thoracic surgery where he underwent a diverticulectomy with stapling and additional upper oesophageal myotomy. The surgical intervention was done with a left-lateral open surgical approach, after assessing operation conditions with intraoperative oesophagoscopy.

The patient was hospitalized for seven days, with daily follow-up for leakage/infection at the incision site and for integrity of the repair. Oral nutrition was initiated on day five of operation. A control visit was scheduled for one month after discharge. At the control visit, he was free of all symptoms and weighed 60 kg.

## Discussion

Together with the changing world, there is a substantial improvement and availability in high-tech diagnostic/therapeutic facilities. Health systems are always changing too. Today’s doctors have adapted themselves to the new conditions by changing their roles towards more high-tech/low touch behaviours. One important reason for missing the diagnosis in our patient was probably the technology-oriented behaviour of the first gastroenterologist. Some liability can also be attributed to the current performance-based health system in Turkey, leading to congested secondary/tertiary care units [8].

Prior literature has pointed to the difficulty of management when the patient is a doctor himself [9–12]. Although most of those papers advise doctors on how to approach doctor-patients, the patient in our case had significant responsibility for the delay of care.

Perspectives may change when a doctor becomes a patient. Alterations of treatment do not necessarily lead to a better outcome and could even be counterproductive [11]. Hence, the focus of therapy should strictly be on the nature of the ailment, rather than the profile of the patient. It has been said that the doctor is never a satisfactory patient; he attempts to be his own doctor unless the attending physician is very strong-minded, and the sick doctor too ill to criticise [10]. It is, therefore, a great mistake on the part of a doctor who treats a colleague to think that the sick man must, because he is a physician, be regarded as in some way different from an ordinary patient. There are obstacles such as ‘corridor consultations’ and deficiencies in occupational health services and primary healthcare services for patients who are physicians themselves. Doctor-patients tend to deny their symptoms, have fears about confidentiality, and may diagnose themselves and self-prescribe which may lead to deficient care-seeking [12]. There is also significant uncertainty in the attending doctors treating a colleague [9].

The only known curative treatment for ZD is surgery [13]. It is stated that the presence of the diverticular pouch is already a surgical indication [3]. Although the patient benefited from the operation, an earlier intervention could prevent the weight loss and unnecessary suffering.

Some studies revealed the relationship between gastroesophageal reflux disease and ZD, considering that the acid reflux can ascend theoretically to the pharyngeal level, determining mucosal injury at the Killian triangle and cricopharyngeal muscle hypertrophy. An incidence of up to 72% of gastroesophageal reflux was found in patients with ZD. The pathologic relationship between these clinical entities can only be speculated. Nevertheless, long-term administration of proton pump inhibitors in patients with or without surgical treatment for ZD can be justified [14].

From the patient’s symptoms, cough, dysphagia, weight loss, and exacerbating factors such as drinking coffee can be observed in other gastrointestinal conditions such as GERD and malignancies [6,15]. However, undigested food coming into the mouth and the typical fluid sound produced by swallowing should not be confused with other entities, given that the physician is attentively listening and making a differential diagnosis.

Although characteristic symptoms of ZD are less frequently present in patients compared to dyspepsia, gastroesophageal reflux disease, or irritable bowel syndrome, its presence should always be taken into consideration during differential diagnosis of diseases of the digestive tract [5]. Family physicians are expected to be able to differentiate between a wide range of illnesses, with less in-depth knowledge in rare conditions, but to be highly competent in managing common diseases [16].

Although a relatively rare condition, ZD should be easily recognized in primary care conditions due to its distinctive history and presentation, in particular when there are both digestive as well as respiratory symptoms, as in this case. There is a need for a high index of suspicion for ZD in patients with dysphagia, cough, and aspiration [17]. Affecting more than 20% of the population [15], GERD is a much more common gastrointestinal condition. Hence, it is no surprise that this patient was initially treated as such a case. Even Rumination syndrome [18] should be kept in mind for patients complaining of regurgitating undigested food.

## Conclusion

This case demonstrates once more the importance of history taking and follow-up in medical care. Partially due to the lack of developed technology, doctors in the past were forced to apply more touch-oriented medical models. This model has become obsolete in our developed world. However, in our opinion, there is a need to merge today’s high-tech medicine with the sensory doctor of the past, who used to listen and touch his patients instead of ordering instant tests. Doctor-patients should be met in the same way as other patients, but also the doctors themselves should be trained on how to behave in case of having a health condition.
